# UK experience of ofatumumab in recurrence of focal segmental glomerulosclerosis post-kidney transplant

**DOI:** 10.1007/s00467-021-05248-9

**Published:** 2021-08-12

**Authors:** Ben C. Reynolds, Angela Lamb, Caroline A. Jones, Pallavi Yadav, Kay S. Tyerman, Colin C. Geddes

**Affiliations:** 1 Department of Pediatric Nephrology, Royal Hospital for Children, Glasgow, UK; 2 Department of Pediatric Nephrology, Alder Hey Children’s NHS Trust, Liverpool, UK; 3 Department of Pediatric Nephrology, Leeds Children’s Hospital, Leeds, UK; 4 Department of Nephrology, Queen Elizabeth University Hospital, Glasgow, UK

**Keywords:** Transplant outcomes, Steroid-resistant nephrotic syndrome, Immunosuppression, Plasmapheresis

## Abstract

**Background:**

Steroid-resistant nephrotic syndrome (SRNS), commonly caused by focal segmental glomerulosclerosis (FSGS), is associated with progression to stage 5 chronic kidney disease, requirement for kidney replacement therapy and a risk of disease recurrence post-kidney transplantation. Ofatumumab (OFA) is a fully humanised monoclonal antibody to CD20, with similar mechanisms of action to rituximab (RTX).

**Methods:**

We report a case series of seven UK patients (five paediatric, two adult), all of whom developed FSGS recurrence after kidney transplantation and received OFA as part of their therapeutic intervention. All also received concomitant plasmapheresis. The 2-year outcome of these seven patients is reported, describing clinical course, kidney function and proteinuria.

**Results:**

Four patients (all paediatric) achieved complete urinary remission with minimal proteinuria 12 months post-treatment. Three of those four also had normal graft function. Two patients showed partial remission—brief improvement to non-nephrotic proteinuria (197 mg/mmol) in one patient, maintained improvement in kidney function (estimated glomerular filtration rate 76 ml/min/1.73 m^2^) in the other. One patient did not demonstrate any response.

**Conclusions:**

OFA may represent a useful addition to therapeutic options in the management of FSGS recurrence post-transplantation, including where RTX has shown no benefit. Concomitant plasmapheresis in all patients prevents any definitive conclusion that OFA was the beneficial intervention.

**Supplementary Information:**

The online version contains supplementary material available at 10.1007/s00467-021-05248-9.

## Introduction

Steroid-resistant nephrotic syndrome (SRNS) is defined as proteinuria > 200 mg/mmol creatinine, hypoalbuminaemia and clinical oedema with no remission of proteinuria following 4 weeks of high-dose (2 mg/kg) corticosteroids, or subsequent loss of steroid responsiveness over time. In childhood, focal segmental glomerulosclerosis (FSGS) is the most common histological finding in SRNS. Progression to stage 5 chronic kidney disease (CKD 5) and requirement for kidney replacement therapy is common in FSGS—43–94% of patients enter CKD 5 within 10 years of diagnosis, depending on immunosuppressant response [1].

Various immunosuppressive agents trialled in FSGS include calcineurin inhibitors (ciclosporin, tacrolimus), mycophenolate mofetil and the chimeric anti-CD20 monoclonal antibody, rituximab (RTX); calcineurin inhibition is recommended as first-line treatment in pre-dialysis patients. FSGS carries a risk of disease recurrence post-kidney transplant. The risk of disease recurrence is increased in patients presenting at a younger age, with initial steroid sensitivity, or no identified genetic mutations—all felt to represent an immunological aetiology being more likely, and no response to other trialled immunosuppressive therapies [2, 3], and much lower where a secondary cause is identified. Confirmation of a genetic aetiology or complete steroid resistance does not preclude recurrence. Recurrence may occur within hours of transplantation or months to years later. Management of FSGS recurrence post-transplant is not standardised, with no robust evidence to support any particular management stratagem in all patients—likely partly attributable to the disease heterogeneity itself [4]. Current accepted strategies include the use of plasmapheresis (PLEX), optimisation of calcineurin inhibitors and depletion of B cells using a monoclonal antibody, most commonly RTX [5]. Many hypotheses presume an as-yet unidentified circulating factor [2], hence the rationale for PLEX in disease recurrence post-transplant, which has a reasonable body of literature to support its use [6]. PLEX is reportedly more effective in early recurrence and in paediatric patients [7, 8].

Recommendations on dose and duration of PLEX are weaker, although current international guidance suggests three daily exchanges then six further exchanges over 2 weeks, or a slow weaning course over 9 months [6]. More intensive PLEX regimens have been used with some success [9, 10]. Additional immunosuppression is recommended, as PLEX serves only to remove the presumptive circulating ‘permeability factor’. Limited evidence exists surrounding the use of ciclosporin, cyclophosphamide, intravenous immunoglobulin, and more esoteric therapies such as galactose and bone marrow mesenchymal stem cell infusions [11–13].

With the assumption that the circulating factor is B-cell derived/influenced, RTX has been used to treat post-transplant recurrence, with some success [14]. RTX is a chimeric humanised murine monoclonal antibody directed against the large extracellular loop of CD20, commonly expressed on B cells, and used for B-cell depletion (although ineffective against pre-B cells and fully differentiated plasma cells, neither of which express CD20). Usage of RTX includes proven benefit in steroid-sensitive nephrotic syndrome and some limited benefit in SRNS with a reported 20–44% response rate [15]. RTX was first used in a patient with post-transplant FSGS recurrence as part of chemotherapy for post-transplant lymphoproliferative disease with the unexpected benefit of disease remission [16]. Subsequently, RTX has been used peri-operatively at the time of transplant [17] and in management of recurrent FSGS [18] with some success. Response to RTX appears more likely in paediatric patients [18]—whether this represents a different aetiology in paediatric FSGS or intrinsic differences in younger patients’ response is unknown.

Ofatumumab (OFA) is a fully humanised monoclonal antibody directed against CD20, binding both the small and large extracellular loop [19]. Being fully humanised reduces the likelihood of reaction to the murine elements: use of OFA in RTX hypersensitivity is a recognised indication [20]. Whether OFA offers therapeutic superiority to RTX is unclear. Much of the lymphoma literature suggests no benefit [21]. A randomised controlled trial investigating low-dose OFA versus RTX in the treatment of steroid-dependent NS pre-transplant, admittedly quite a different disease entity, was terminated early due to futility [22].

The first case series describing the use of OFA in five patients with SRNS, one of whom had recurrence post-transplant [23], demonstrated remission of recurrent disease. Following the publication of that report and awareness amongst paediatric nephrologists, OFA has been reported with variable efficacy in several case reports and a small case series [24]. We summarise what we believe to be the known UK usage of OFA in paediatric kidney transplant patients, with additional reporting of two adult patients in our local unit, all of whom developed recurrent FSGS post-kidney transplantation.

## Methods

All 13 UK paediatric nephrology centres were asked to inform the authors if they had used, or were aware of the use of, OFA for post-transplant recurrence through an electronic request via the British Association of Pediatric Nephrology (BAPN) in 2018, with additional informal communications with each centre by the lead author. For each case identified, data was collected retrospectively on disease course pre-transplant including any identified genetic mutation, initial steroid sensitivity, time to CKD 5, time on dialysis and other immunosuppressive therapies used. Post-transplant data was collected either retrospectively or prospectively and included baseline immunosuppression, time to disease recurrence, whether dialysis was required, use and intensity of PLEX, indication, timing and dose details of OFA, and longitudinal measurements of serum creatinine, serum albumin, urinary protein/creatinine ratio and estimated glomerular filtration rate (eGFR, estimated using the bedside Schwarz equation). Data was collected for up to 2 years post-transplant or until another intervention after OFA occurred.

Complete urinary remission was defined as improvement in proteinuria to a UPCR of < 20 mg/mmol creatinine. Partial urinary remission was defined as improvement in proteinuria to non-nephrotic range, i.e. < 200 mg/mmol creatinine. Normalisation of graft function was defined as an increase in estimated GFR from < 90 to ≥ 90 ml/min/1.73 m^2^. Improvement of graft function was defined as an increase in eGFR of ≥ 10 ml/min/1.73 m^2^ after drug administration.

## Results

The authors were personally involved with treatment of three patients in two UK centres at the time of the BAPN request in 2018. Although no additional patients were identified following that request or by direct communication with centres, three further patients were discussed with the authors, and subsequently received OFA (two adult within our own unit and one further paediatric case in another centre). A further case in our own centre also received OFA following the survey. No additional paediatric cases have received OFA to our knowledge. Thus, seven patients from three paediatric centres and one adult centre are included.

In the first UK paediatric case (case 5 below), OFA was administered as the patient had prior hypersensitivity to RTX. In two cases, OFA was used after RTX administration post-transplant with ongoing evidence of disease recurrence. Two cases had previously received RTX when pre-dialysis without effect, so OFA was used in preference post-transplant. In the other two cases, OFA was used as the initial monoclonal agent after detailed discussion with the family regarding the unknown efficacy for this indication but suggestion (at the time) of clinical superiority over RTX.

The dose of OFA administered for all but case 5 was 300 mg/1.73 m^2^ as an initial dose, followed by five weekly doses of 2000/1.73 m^2^ over a total of 6 weeks, unless clinical circumstances led to delayed administration. The dosing schedule used is provided in Supplementary Appendix 1.

**Fig. 1:**
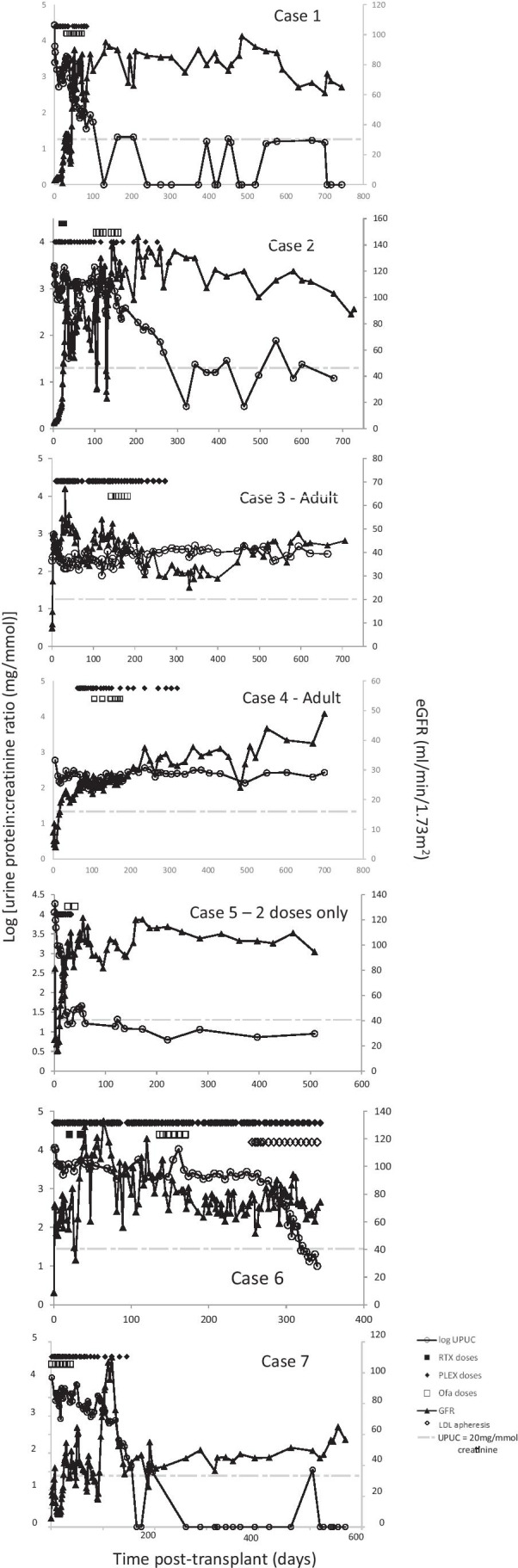
Longitudinal timeline for all patients demonstrating change in proteinuria and estimated GFR, relating to administration of plasmapheresis, rituximab (where relevant) and ofatumumab. The light grey dotted line represents a urinary protein/creatinine ratio of 20 mg/mmol creatinine

Notable case details are summarised in Table 1, including initial disease course, potentially relevant transplantation data, details on recurrence and therapies, and biochemical parameters. Figure 1 summarises individual patient treatments with longitudinal changes in eGFR and proteinuria. Each case is briefly summarised below.

**Table 1:** Case details summarising clinical characteristics, transplant details, disease recurrence and treatments administered

Case	1	2	3	4	5	6	7
Recurrence risk							
FSGS	Yes	Yes	Yes	Yes	Yes	Yes	Yes
Male	Yes	No	Yes	Yes	No	Yes	Yes
Age at presentation (years)	1.5	6	18	43	2	2	2
Genetic mutation	None	Unlikely*	None	Not tested	None	None	None
Early pre-transplant steroid sensitivity	Yes	Yes	No	Yes	Yes	Yes	No
No immunosuppression response	None	CyA	None	CyA	CyA, Tac, RTX, CYP	None	None
Transplant (Tx) details							
Age at transplantation (years, months)	7y 4 m	11y 11 m	23	52	14y 4 m	6y 2 m	4y 6 m
DD vs. LD	DBD	DBD	L(R)D	DCD	LD	L(R)D	L(R)D
Warm ischaemic time (min)	53	42	32	15	47	39	57
Cold ischaemic time	25 h 6 min	22 h 5 m	2 h 57 min	10 h 2 min	9 h 14 min	85 min	4 h 57 min
HLA mismatch	111	111	100	010	110	011	111
Immunosuppression	TWIST	TWIST	TWIST	TWIST	Tac/Pred/Aza	TWIST	TWIST
Recurrence details							
Onset of UPUC > 200 mg/mmol (days post-transplant)	0	0	2	7	0	0	0
Histological confirmation of recurrence	Yes	Yes	Yes	Yes	No	No	Yes
Dialysis requirement post-transplant	Yes	Yes	No	No	Yes	Once	Yes
Duration of dialysis requirement (days)	42	17	–	–	4	1	27
Treatment details							
Time 1st PLEX (days post-Tx)	3	5	19	63	3	1	1
UPUC pre-ofatumumab	3235	2907	132	162	16	2155+	10,905
eGFR pre-ofatumumab	34	99	48	28	91	93	6
Time 1st ofatumumab (days post-Tx)	31	104	143	106	27	138	1
Time to complete 6 doses	7 weeks	9 weeks	7 weeks	7 weeks	2 doses only	7 weeks	7 weeks
Time on PLEX (months)	3	9	10	9	1	6	5
Other treatments	IVMP	RTX, re-stent	None	None	IVMP	IVMP LDL apheresis	IVMP
Follow-up details							
Time post-Tx to UPUC < 200 (days)	58	204	17	14	20	NA	132
Time post-Tx to complete remission UPUC < 20 mg/mmol (days)	128	321	NA	NA	26	328	165
UPUC 3 months post-OFA (mg/mmol creat)	55	123	363	201	12	2661+	225
eGFR 3 months-post OFA (ml/min/1.73 m^2^)	76	126	30	29	92	76	52
UPUC 6 months post-OFA (mg/mmol creat)	< 3	< 3	366	310	6	25+	< 3
eGFR 6 months post-OFA (ml/min/1.73 m^2^)	68	130	33	38	114	72	39
UPUC 12 months post-OFA (mg/mmol creat)	< 3	14	197	263	AWAITED	NA	< 3
eGFR 12 months post-OFA (ml/min/1.73 m^2^)	90	113	45	44	103	NA	45
6 month serum albumin (g/dl)	35	30	37	31	38	24	43

*Aza* azathioprine, *CyA* ciclosporin, *CYP* cyclophosphamide, *DBD* donation after brain death, *DCD* donation after cardiac death, *DD* deceased donor, *eGFR* estimated glomerular filtration rate, *FSGS* focal segmental glomerulosclerosis, *HLA* human leukocyte antigen, *IVMP* intravenous methylprednisolone, *L(R)D* living (related) donor, *NA* not available, *PLEX* plasma exchange, *RTX* rituximab, *Tac* tacrolimus, *TWIST* basiliximab, tacrolimus, mycophenolate mofetil and prednisolone, *UPUC* urine protein/creatinine ratio

^*^Genetic analyses identified autosomal recessive heterozygous mutation in PTPRO not felt to be pathogenic

+ Urinary albumin/creatinine ratio reported for case 6

Complete urinary remission occurred in three cases, all paediatric. Partial urinary remission to non-nephrotic range proteinuria occurred in one adult case, but was brief and rapidly reverted to nephrotic range. No urinary response was seen in two cases, one adult and one paediatric. One case had nephrotic range proteinuria with complete urinary remission during PLEX; this was maintained following OFA and rapid cessation of PLEX. Graft function normalised in one paediatric case following OFA. Two cases had normal graft function at the time of administration but ongoing nephrotic range proteinuria. Two cases (one paediatric, one adult) demonstrated improvement in graft function after OFA administration, but did not achieve a normal eGFR. No benefit was seen on graft function in two patients (one paediatric, one adult).

## Case 1

A 7-year-old African boy received a deceased donor (DD) kidney transplant, 5 years after diagnosis. Pre-dialysis, RTX administration had not been beneficial. Disease recurrence was immediate, with nephrotic range proteinuria and graft dysfunction necessitating haemodialysis 1 day post-operatively. PLEX was commenced on day 4, delivering five exchanges in the first week, then thrice weekly for 1 week, with a reducing frequency thereafter. OFA was commenced on day 31 post-transplant. The initial infusion was associated with itch, which resolved when reducing the rate of infusion. Subsequent doses were uncomplicated. Kidney function improved and proteinuria declined from 4 and 6 weeks post-transplant, respectively.

An episode of possible rejection 2 months post-transplant was treated with 3 days of intravenous methylprednisolone; subsequent biopsy confirmed FSGS recurrence with no evidence of acute rejection. PLEX was discontinued after 3 months of treatment due to loss of central venous access. Complete urinary remission was achieved 8 months post-transplant and is negligible 3 years post-transplant. Graft function was 80–90 ml/min/1.73 m^2^ up to 18 months post-transplant, but has deteriorated recently in association with antibody-mediated rejection.

## Case 2

An 11-year-old Caucasian female received a DD kidney transplant, with dilatation of the proximal donor ureter. Delayed graft function required reinstatement of peritoneal dialysis from day 1. Immediate nephrotic proteinuria suggested recurrence, so PLEX was commenced day 3 post-transplant, three exchanges weekly for 3 weeks then reducing in frequency. Recurrent FSGS was confirmed histologically 2 weeks post-transplant, with the administration of RTX 750 mg/m^2^ twice 1 week apart at that time. Three months post-transplant, nephrotic range proteinuria persisted so OFA was administered. B Cells were not checked prior to administration, so it is unknown whether early reconstitution had occurred. An acute kidney injury following the first dose was due to ureteric ischaemia, managed with re-insertion of ureteric stent. This delayed subsequent dosing with OFA by 2 weeks. No other doses were associated with adverse events. Proteinuria became non-nephrotic 6 months post-transplant and demonstrated complete remission 6 months after OFA was completed. PLEX was discontinued 9 months post-transplant. She currently has normal kidney function (eGFR 91 ml/min/1.73 m^2^) and negligible proteinuria 3 years post-transplant.

## Case 3

A 23-year-old Caucasian male received a living related donor (LRD) transplant, with immediate graft function. Nephrotic range proteinuria developed on day 2 with a reduction in the rate of decline of serum creatinine. Alternate day PLEX improved proteinuria (although still within nephrotic range) and kidney function—this improvement was not maintained when exchange frequency was reduced. Biopsy demonstrated widespread foot process effacement but normal light microscopy appearance. Frequency of PLEX was maintained at thrice weekly. OFA was considered, as RTX was ineffective pre-dialysis, and commenced 5 months after transplant. He developed a mild rash during the first infusion but no other adverse effects were noted during the remaining infusions. A brief improvement in proteinuria to 97 mg/mmol 3 weeks after completion of OFA was not maintained, with rapid recurrence of nephrotic range proteinuria. No improvement in graft function was noted, although this stabilised at 50–60 ml/min/1.73 m^2^.

## Case 4

A 53-year-old Caucasian male received a DD kidney transplant. Graft function was immediate, but did not improve as expected, and non-nephrotic range proteinuria persisted, prompting a trial of PLEX. Non-nephrotic range proteinuria (82–173 mg/mmol) continued, as did graft impairment (eGFR 26–30 ml/min/1.73 m^2^), so additional immunotherapy was considered. Given the absence of response to RTX pre-transplant, OFA was administered. No adverse effects were noted. There was no urinary response; proteinuria deteriorated and entered the nephrotic range. Graft function improved slowly over time, from 20 to 30 ml/min/1.73 m^2^ at the time of initial OFA administration to 40–50 ml/min/1.73 m^2^ 12 months later.

## Case 5

A 14-year-old Caucasian female received an altruistic living unrelated transplant. Nephrotic range proteinuria was evident by day 2 post-transplant, with PLEX commenced on day 3. Graft dysfunction continued with an early biopsy demonstrating acute tubular injury. Hemodialysis was required between day 7 and day 11 post-transplant. Five sessions of PLEX over 2 weeks were followed by four sessions over 3 weeks. The patient had previously experienced a serum-sickness-like reaction to RTX, so received two doses of OFA 700 mg (approximately 750 mg/1.73 m^2^) on days 25 and 32 post-transplant. Graft function and proteinuria had normalised prior to OFA administration. PLEX was discontinued after the first dose of OFA. The patient maintained complete urinary remission and normal graft function for 18 months post-transplant. Subsequent graft dysfunction was associated with medication non-concordance, with no associated proteinuria.

## Case 6

A 6-year-old Caucasian male received a LRD kidney transplant. Graft function was evident at 24 h with immediate nephrotic range proteinuria, leading to treatment with RTX and PLEX, initially 10 doses in 2 weeks and subsequently 3 doses each week. An episode of possible rejection was treated with IV methylprednisolone. Proteinuria remained in the nephrotic range, prompting administration of OFA 4 months following transplantation, with no adverse effects observed during infusions. No change in proteinuria or graft function was seen in the 3 months following OFA administration. Low-density lipoprotein apheresis was subsequently commenced 8 months post-transplant, with complete remission of proteinuria and maintained graft function.

## Case 7

A 4-year-old Caucasian male received a LRD kidney transplant. Graft function was immediate, with nephrotic-range proteinuria evident at 12 h post-transplant. PLEX was commenced days 1 and 2 post-transplant, then 8 sessions over 2 weeks, weaning to 3 sessions/week for 3 weeks, 2 sessions/week for 4 weeks, weekly for 4 weeks, then finally every 2 weeks for 4 sessions. OFA was commenced day 1 post-transplant; no adverse reactions were noted. FSGS recurrence was confirmed histologically 6 weeks post-transplant. No response was seen initially—nephrotic range proteinuria persisted and eGFR was 40 ml/min/1.73 m^2^ at 3 months post-transplant. A suspected episode of rejection was treated with pulsed IV methylprednisolone—this led to dramatic improvement in both proteinuria and graft function. Histology demonstrated no rejection; only evidence of FSGS recurrence. Renewed steroid sensitivity was suspected, so oral steroids were continued on a slowly weaning course. At 12 months post-transplant, graft function improved to 60 ml/min/1.73 m^2^ and complete urinary remission was achieved.

## Discussion

We describe seven patients, five paediatric and two adult, who received OFA as part of their therapeutic strategy for recurrent FSGS post-kidney transplant, with variable efficacy. This case series represents one of the larger series in the literature, provides medium-term follow-up to 2 years post-transplantation, and reports both successful and unsuccessful outcomes. This is the first report of OFA in adult FSGS recurrence. No patients received OFA in isolation—all had other immunomodulatory therapies ongoing, including plasmapheresis.

Reduction in proteinuria was evident in all paediatric patients, with four/five demonstrating complete urinary remission between 6 and 9 months post-transplant, and all in complete urinary remission by 12 months. Introduction of steroids in one patient, and usage of lipid apheresis in another, was closely associated with urinary remission in two cases, so causation by OFA cannot be assumed. A maintained response was not seen in either of the adult patients receiving OFA.

Improvement in graft function was only seen in 2/7 patients. Two paediatric patients had good graft function prior to OFA administration. Graft function improved to a normal eGFR in one of the paediatric patients and improved over time (though still impaired) in one adult and one paediatric patient. No benefit on graft function was seen for two patients.

Pediatric FSGS is associated with a much greater risk of disease recurrence than FSGS occurring in adult patients, with young age of onset a key risk factor [18]. In one series, 86% of paediatric patients had disease recurrence, compared to 35% of adult patients. However, younger patients may have a different disease entity or responsiveness compared to older patients [18, 25]. Adult remission rates from recurrence occur in ~ 50% of patients [5]. Determining the remission rate in children is more difficult, due to publication bias, lack of large case series and relative rarity of the diagnosis. No literature exists on OFA in adult FSGS, so it is unknown whether the absence of response seen in our two patients is as expected. The modest but evident improvement in most paediatric patients in this series, compared to adult patients, again suggests that paediatric FSGS may have a more modifiable disease course.

Six of the seven patients received a short course of prednisolone immediately post-transplantation. Although there is a recognised increased risk of disease recurrence associated with steroid minimisation regimens for some immunologically mediated renal conditions, there is no definitive support that this is the case for FSGS. Two adult cohorts totalling 148 patients and one paediatric cohort of 25 patients did not demonstrate increased recurrence risk with short steroid regimens [26–28].

There are several limitations to this paper. Most importantly, all patients also received concomitant PLEX, which is a major confounding factor and may also account for improvement in proteinuria and graft function. Although this confounding effect of ongoing PLEX cannot be excluded, in two patients with clinical response, one had a rapid cessation of plasmapheresis following OFA due to technical issues (case 1), and the second had received a lengthy trial of plasmapheresis with no clear benefit prior to OFA administration (case 2). We believe OFA directly benefited these patients.

Other limitations include the significant case heterogeneity, particularly with other immunosuppression. One patient received LDL apheresis, and a second was re-commenced on corticosteroids after OFA administration. One patient had normalisation of graft function and proteinuria prior to OFA administration so determining benefit is impossible. Two patients received RTX within 6 months prior to OFA administration—B-cell data is unavailable so it is unknown whether either patient had reconstituted by the time of OFA infusion. It is plausible that ongoing improvement in one of these two patients could be attributable to RTX, although the second patient had no change in clinical course until LDL apheresis was introduced. One patient demonstrated marked improvement after the administration of high-dose corticosteroids, having previously been steroid resistant—again, clinical improvement may not be attributable to the OFA. The total number of patients in this series remains small, across several centres—there may have been practice variation in other aspects of transplant care that could also be confounding. The indications for OFA were also varied—previous RTX hypersensitivity, previous failure of RTX pre- or post-transplant, and latterly, used in preference to RTX due to previous perceived patient improvement.

OFA is a fully humanised anti-CD20 monoclonal antibody, initially used in patients with chronic lymphocytic leukaemia, and licensed for patients with a recognised sensitivity/anaphylaxis to RTX administration. OFA has since been reported in a variety of different conditions, most commonly in patients unable to tolerate RTX, including anti-neutrophil cytoplasmic antibody-associated vasculitis, IgA nephropathy and lupus nephritis [29–31]. Similar to RTX, the first usage in NS was in a patient with both resistant NS and leukaemia [32]. Several reports now exist of OFA usage in both steroid-sensitive and steroid-resistant NS, where either there was absence of response to RTX or patients had hypersensitivity reactions [20, 23, 33]. An open-label randomised controlled trial comparing RTX and OFA in paediatric steroid-dependent NS ended early due to futility, although notably used a far lower dose of OFA than previous reports [22]. Disease remission of SRNS has occurred in a significant majority of reported patients [23], although this may be subject to publication bias. The same case series also included a desensitisation protocol, as four of that centre’s five patients demonstrated hypersensitivity reactions [23].

In our series, no major infusion reactions were noted in any patient. One patient had itch during the first infusion, a second patient developed urticaria which rapidly settled—neither required any additional intervention other than symptomatic treatment and tolerated further infusions with no complication. All patients completed the intended course of OFA (in case 5, OFA was used at lower dose). No major infective episodes were identified during the initial administration or in the 2 years subsequently. One patient developed transplant ureter ischaemia and subsequent necrosis, attributed to pre-existing anatomical abnormalities noted at the time of anastomosis—although a correlation with OFA cannot be excluded. The overall adverse effect profile of OFA appears acceptable in both the chronic lymphocytic leukaemia population [34] and in children with NS [35] with infusion reactions and infection being most commonly reported.

Usage of OFA for post-transplant recurrence has now been reported in several case reports and small case series [24, 36–38]. Colucci et al. reported two patients demonstrating a complete and partial remission [37]; Solomon et al., a patient with partial remission [36]. All three patients had initially received RTX with no maintained improvement. Kienzl-Wagner reported pre- and post-transplant treatment with OFA and PLEX in a patient receiving their second graft, with partial remission achieved [38]. Bernard et al. reported one patient with partial remission following OFA in a patient receiving their second renal graft, i.e. particularly high risk for disease recurrence [39]. In the last two patients, disease recurrence was associated with B-cell reconstitution and repeated OFA dosing maintained a partial remission. The same group reported the outcome of six patients (including the patient above), three achieving partial remission and three having no response [24]. Two different dose strategies were used, patients had a median age of 16 years, and administration of OFA was performed between 1 month and 3 years post-transplant, so a significant patient heterogeneity was evident. These papers support our findings that OFA may have a role where there has been a lack of efficacy from RTX, but that it does not benefit all patients, and those benefits may be modest.

The mechanism of action of OFA in FSGS recurrence requires further elucidation. Whether B-cell depletion is responsible for disease remission is complicated by demonstration of direct binding of RTX to podocytes [17], although the validity of this has been questioned and an effect on Interleukin-4 proposed as an alternative mechanism [40]. However, RTX has a demonstrable improvement on proteinuria in animal models of chemical (adriamycin) induced nephropathy [41], i.e. unrelated to immunological aetiologies. OFA binds to a different CD20 epitope with greater avidity and induces more cell-dependent cytotoxicity than RTX [42] with demonstrably better outcomes compared to RTX in murine models of lymphoma [43]. There has been no work to date examining OFA binding sites on the podocyte. Greater avidity, alternative binding or a differential effect on B-cell function may all contribute to explaining OFA response in patients with RTX resistance.

Other novel anti-CD20 monoclonal agents, including obinutuzumab and ocrelizumab, have been licensed for other indications in adult patients. Whether these will have similar safety and efficacy profiles in children is unknown, and molecular differences preclude assuming the same responses, although obinutuzumab may be predicted to have similar efficacy. OFA was withdrawn commercially from the European Union in 2019, although it is still available in the USA and on an individual compassionate basis by application—the authors have used this route successfully for UK patients. A subcutaneous preparation of OFA for adult patients with multiple sclerosis has recently been approved.

## Conclusion

We report a small case series of seven patients receiving therapeutic OFA for recurrent FSGS post-kidney transplantation. Four of five paediatric cases demonstrated improvement, with either complete or partial remission. Neither adult patient demonstrated an objective maintained response. All patients received concomitant plasmapheresis, which is a crucial limitation and prevents attribution of response to OFA. Recognised adverse effects from OFA were minimal and did not prevent further dose administrations. The mechanisms of action of B-cell depletion in mitigating FSGS recurrence remain unclear. Whether OFA offers any superiority to RTX as an adjunct treatment is unknown, but it appears a safe alternative.

## Supplementary Information

Below is the link to the electronic supplementary material.

Supplementary file1 (DOCX 19 KB)
