# Supplementary material for: UK experience of ofatumumab in recurrence of focal segmental glomerulosclerosis post-kidney transplant
Source: Pediatr Nephrol. 2021 Aug 12;37(1):199–207. doi: 10.1007/s00467-021-05248-9 (PMC8674165; doi:10.1007/s00467-021-05248-9)
Supplement: Supplementary file 1 — Supplementary file1 (DOCX 19 KB) [file 467_2021_5248_MOESM1_ESM.docx]

UK experience of ofatumumab in recurrence of steroid-resistant nephrotic syndrome post-kidney transplant

Journal: Pediatric Nephrology

Ben C Reynolds PhD^1^, Angela Lamb BSc^1^, Caroline A Jones MD^2^, Pallavi Yadav MD^3^, Kay S Tyerman FRCPCH^3^, Colin C Geddes FRCP(Glas)^4^

Corresponding Author: Ben C Reynolds

Dept of Pediatric Nephrology

Royal Hospital for Children

1345 Govan Road

Glasgow

G51 4TF

United Kingdom

Tel: +44 0141 451 6563 ORCID ID: 0000-0002-2928-7493

E-mail: [ben.reynolds@ggc.scot.nhs.uk](mailto:ben.reynolds@ggc.scot.nhs.uk)

Supplementary Appendices

Contents:

Appendix 1: Ofatumumab administration schedule ..................... 3

**Supplementary Appendix 1:**

**– Ofatumumab dosing schedule**

Day-case admission is required, but no specific dietary requirements or lifestyle changes prior to/during administration. Weekly dosing schedule.

**Regimen**

- Dose 1 : I.V. Ofatumumab 300 mg/1.73 m^2^ – test dose
- Dose 2 : I.V. Ofatumumab 2000 mg/1.73 m^2^
- Dose 3 : I.V. Ofatumumab 2000 mg/1.73 m^2^
- Dose 4 : I.V. Ofatumumab 2000 mg/1.73 m^2^
- Dose 5 : I.V. Ofatumumab 2000 mg/1.73 m^2^
- Dose 6 : I.V. Ofatumumab 2000 mg/1.73 m^2^

**PRE-MEDICATION DRUGS**

Oral paracetamol (acetaminophen) 15 mg/kg (max. 1gm) orally – 60 minutes prior to infusion

IV Methylprednisolone 60 minutes prior to Ofatumumab infusion:

1–5years - 50mg 6 years and above – 100mg

Chlorphenamine orally 60 minutes prior to infusion:

1–5 years – 1mg 6–12 years – 2mg >12 years – 4mg

**INFUSION THERAPY**

**First infusion – dose 1**

- **I.V. Ofatumumab 300 mg in 1000 mls normal saline (sodium chloride 0.9%)**

To be infused as follows:

| **300mg in 1000mls solution** | Rate : |  |
| --- | --- | --- |
| 1^st^ 30 minutes | 12 ml/hour | If tolerated increase |
| 2^nd^ 30 minutes | 25 ml/hour | If tolerated increase |
| 3^rd^ 30 minutes | 50 ml/hour | If tolerated increase |
| 4^th^ 30 minutes | 100 ml/hour | If tolerated increase |
| 5^th^ 30 minutes | 200 ml/hour | If tolerated increase |
| 6^th^ 30 minutes | 400 ml/hour | Maximum rate |

**Second and subsequent infusion – dose 2–6 on a weekly basis**

- **I.V. Ofatumumab 2000 mg in 1000 mls normal saline (sodium chloride 0.9%)**

NB: If the first infusion has been completed without severe adverse reaction the remaining infusions should be administered over 4 hours

To be infused as follows:

| **2000 mg in 1000 ml solution** | Rate : |  |
| --- | --- | --- |
| 1^st^ 30 minutes | 25 ml/hour | If tolerated increase |
| 2^nd^ 30 minutes | 50 ml/hour | If tolerated increase |
| 3^rd^ 30 minutes | 100 ml/hour | If tolerated increase |
| 4^th^ 30 minutes | 200 ml/hour | If tolerated increase |
| 5^th^ 30 minutes | 400 ml/hour | Maximum rate |
